# Tuberculosis following kidney transplantation: report of paediatric case

**DOI:** 10.11604/pamj.2015.22.302.7882

**Published:** 2015-11-25

**Authors:** Kamel Abidi, Manel Jellouli, Yousra Hammi, Tahar Gargah

**Affiliations:** 1 Pediatric Nephrology Department, Charles Nicolle Hospital, Tunis, Tunisia

**Keywords:** Pulmonary tuberculosis, renal failure, renal transplantation, child

## Abstract

Recipients of solid organ transplantation are, because of immunosuppressive therapy, at high risk to develop opportunistic infections including tuberculosis (TB). The incidence, clinical manifestations, and optimal diagnostic tests of this disease in this population have not been adequately defined. In this paper, we report a case of 13 year-old boy who developed pulmonary tuberculosis following a second renal transplantation from a deceased donor. The described case points diagnostic difficulties of the tuberculosis disease which are due to insidious and non specific clinical presentation. Also, the treatment is delicate because interaction between immunosuppressive drugs and antituberculosis drugs.

## Introduction

It is well documented that immunosuppressive therapy exposes solid organ recipients to many serious complications including opportunistic infections [1]. In such immunocompromised patients, tuberculosis re-emerged as a serious complication frequently observed with atypical presentation causing delay in diagnosis [2]. The most of published cases were described in adult recipients from endemic area. In children tuberculosis has been rare and the few cases that have been reported [3]. We report here a rare case of pulmonary tuberculosis in a pediatric renal recipient with good outcome after conventional anti tuberculosis therapy.

## Patient and observation

**Figure 1:**
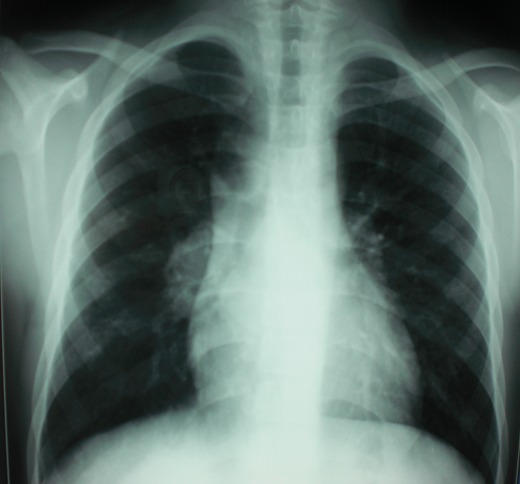
Chest radiography: superior mediastinal enlargement

**Figure 2:**
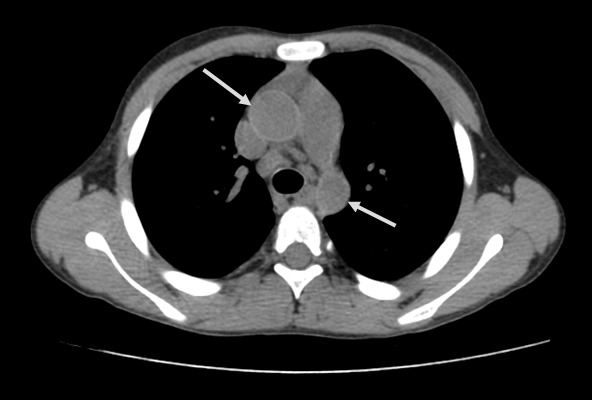
Chest computed tomography: anterior mediastinal mass on right and left main stem bronchi

**Figure 3:**
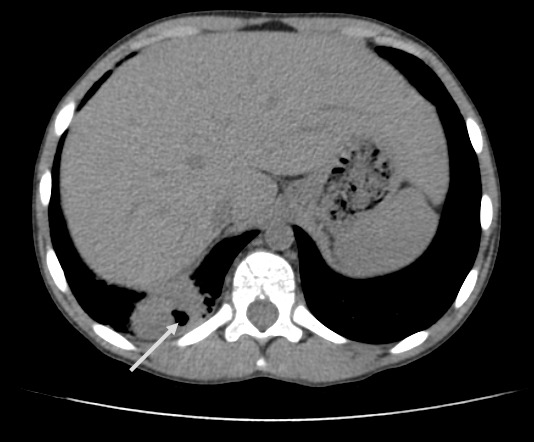
Chest computed tomography: cavitatory pneumonia of right lower lobe

A 13 year-old Tunisian male was admitted in our department of paediatric nephrology on October 2009 with a week-history of fever and asthenia. He was suffering from end-stage renal failure attributed to a vesico-ureteral reflux discovered in 2003. In 2007, the patient had a pre-emptive renal transplant from a living donor. The post-operative course was characterised by an acute ischemia of the transplant due to a venous renal thrombosis that lead to a detransplantation. A regular peritoneal dialysis was initiated on June 2007. A second renal transplantation was performed on June 2009 from a 27 year-old deceased donor. The post operative course was uneventful. The patient was maintained on Prednisone 10 mg/day, mycophenolate mofetil 1500 mg/day, and Cyclosporine 75 mg twice per day with adequate renal function. Three months after transplantation, the patient was hospitalized in our department for fever with asthenia. The physical examination on admission revealed asthenic patient, with temperature of 38.5°C, blood pressure of 110/70 mm Hg, pulse rate of 90 /min, and inspiratory rhonchi with decreased breath sound over both bases. There were no peripheral lymph nodes or hepatosplenomegaly. Laboratory investigations revealed hematocrit of 38%, white blood cell count of 8300/mm^3^, platelet count of 220.000/mm^3^, blood urea nitrogen of 6.8 mmol/l, serum creatinine level of 75 µmol/l, C reactive protein serum level of 88.5mg/l and erythrocyte sedimentation rate of 51 mm/hour. Liver function tests and lipid profile were normal. The protein electrophoresis showed a total protein serum level of 65g/l, (Albumin 33.9g/l, α1 globulin 5.8g/l, α2 globulin 9.6g/l, β1 globulin 3.9g/l, β2 globulin 3.4g/l, γ globulin 8.3g/l). The chest x-ray revealed opacity in the right middle zone and superior mediastinal enlargement (Figure 1). A thoracic CT scan showed cavitatory pneumonia of the right lower lobe and anterior mediastinal mass on right and left main stem bronchi corresponding to lymph nodes (Figure 2 and Figure 3). Regarding the clinical, biological and radiological findings, three diagnoses were suspected: pneumonia, pulmonary tuberculosis and pulmonary aspergillosis. By considering the history of the patient and the fact that our country is endemic, our diagnosis was quickly directed towards pulmonary tuberculosis despite the negativity of tuberculin skin test. The search of mycobacterium tuberculosis by gastric tubing was negative but the diagnosis was made by the study of sputum specimens aspirated during bronchoalveolar lavage that revealed the presence of mycobacterium tuberculosis. In vitro studies showed the organisms to be fully susceptible to all antituberculosis drugs. Four drugs, isoniazid (INH), Rifadine (RIF), Ethambutol(EMB) and pyazolin( PZA) were administered orally with the usual doses. The dose of corticosteroids was increased and that of cyclosporine was adjusted to maintain trough plasma levels in recommended therapeutic range. To prevent the neurological side effects of INH we prescribed a pyridoxine in dose of 50 mg per day. The Outcome under antitubercular therapy has so far been favourable, with prolonged apyrexia and regression of the pulmonary abnormalities. This regimen, maintained during 18 months, was well tolerated without any modification in liver tests and graft function.

## Discussion

The incidence of tuberculosis after solid organ transplantation is higher compared with the general population [1–3]. The use of corticosteroids and immunosuppressive drugs interfere with T-cell-mediated function, resulting in decreased host resistance to infection [4–7]. A longer duration of dialysis before transplantation, a previous history of infection, and a poor socioeconomic class are also reported as risk factors [7]. Tuberculosis generally occurs after 6 months of renal transplantation [5, 6]. In our patient, TB was diagnosed early, three months after a second renal transplant. There was no evidence of contact with individuals having active tuberculosis. The mode of contamination is probably a reactivation of latent infection or a transmission by the donor kidney. The clinical presentation of TB may be unusual and subtle in an immunosuppressed transplant recipient [5]. In every case of prolonged fever and unusual clinical manifestations, tuberculosis should be considered especially in endemic countries [5, 7]. The confirmation of mycobacterium tuberculosis infection is difficult. Moreover, the bacteriologic culture may be negative and the tuberculin skin tests are often negative in the transplanted population [8]. In our patient, the search of mycobacterium tuberculosis by gastric tubing was negative but the diagnosis was made fortunately by the traditional tests: Zeihl-Neelsen staining and culture of sputum specimens aspirated during bronchoalveolar lavage. So, this diagnostic procedure should be considered in the diagnosis of pulmonary tuberculosis in children and adults. It allowed a better rate of diagnosis. New diagnostic tests have been developed recently to improve sensitivity and allow a rapid diagnosis of tuberculosis particularly in patients with culture-negative tuberculosis. The latest technique is especially the blood test measuring the Interferon-gamma released by stimulated T cells [8]. However, it should not be used in the first instance for diagnosis of active TB. Apart from the diagnostic difficulties, TB in transplant patients is a real therapeutic problem. Some authors reported an increased rate of acute rejection and a higher rate of graft loss because of interactions between CyA, prednisone and Rifampicin [5, 6]. An adequate and regular monitoring of immunosuppressive drugs avoids the risk of rejection. The neurotoxicity of cyclosporine is well documented [7] and can be increased by INH; we then prescribe systematically pyridoxine to our patient. The prevention of TB is that difficult to achieve in solid organs transplanted patients. The American Thoracic Society has recommended several years ago, a prophylactic INH administration to Mantoux-positive patients [8, 9]. This strategy, however, is unlikely to be applicable in endemic areas, both because of a high degree of Mantoux positivity in the general population as well as the high frequency of false negative tests among chronic renal failure patients. Routine INH prophylaxis is therefore not recommended in endemic areas.

## Conclusion

The clinical presentation of TB after renal transplantation may be different from that of the general population, which makes diagnosis a challenging task. The variety of immunosuppressive drugs which these patients receive and the consequent suppression of cell-mediated immunity should theoretically place them at an increased risk for developing active tuberculosis. Multiple methods should be considered in the diagnostic approach to tuberculosis since none is totally efficient. Finally, the treatment of tuberculosis must take into consideration the drug toxicity and interactions.
